# The Transfer Effects of Cognitive Training on Working Memory Among Chinese Older Adults With Mild Cognitive Impairment: A Randomized Controlled Trial

**DOI:** 10.3389/fnagi.2019.00212

**Published:** 2019-08-14

**Authors:** Wenqi Weng, Jiaming Liang, Jiang Xue, Tingfei Zhu, Yuxing Jiang, Jiayu Wang, Shulin Chen

**Affiliations:** Department of Psychology, Zhejiang University, Zhejiang, China

**Keywords:** mild cognitive impairment, cognitive training, working memory, transfer effects, maintaining effect

## Abstract

**Objectives**: To explore the transfer effects of cognitive training on working memory among older Chinese adults with mild cognitive impairment (MCI).

**Methods**: Sixty-two MCI participants aged more than 60 years old were recruited by holding recruitment sessions in communities in China [33 for cognitive training, and 29 for mental leisure activities (MLA) control]. Cognitive functions, including working memory, execution function, reasoning ability, verbal ability, ability of daily living, were measured at three time-points (baseline, post-training and 3 months after training).

**Results**: Compared to the MLA control, the cognitive training group showed significant effects in both the trained (working memory) and untrained (execution function and ability of daily living) domains. The effects of cognitive training on overall cognitive function, working memory and daily life ability of daily living of MCI could be maintained for at least 3 months, even without the cognitive training. Besides, complete mediating effects of cognitive training were found in executive function through working memory and working memory in ability of daily living though executive function, which suggests the presence of transfer effect of cognitive training.

**Conclusions**: The present study supported that cognitive training could effectively improve working memory in elders with MCI. The training effects on working memory could transfer to other untrained areas (such as executive function), which also improved the comprehensive ability (ability of daily living). And the effects of training could largely persist for 3 months.

## Introduction

The average age of populations is rising—an irreversible trend all over the world, along with which comes the increasing mental health needs of the elderly. And the subsequent increasing number of Alzheimer’s and related dementias cause a heavier burden to society, which is a significant problem. It is estimated that dementia cases have reached nearly 50 million globally, and the incidence of dementia is predicted to rise to 50%, while the number will reach 75 million by 2030 (Prince et al., 2015). Due to its extremely low rate of successful treatment and great social burden, researchers around the world are eager to find new treatment methods for dementia.

As mild cognitive impairment (MCI) is a pre-phase of Alzheimer’s disease (AD) in most cases (Albert et al., 2011; Vos et al., 2015; Klimova and Maresova, 2017), elderly people and their families are seeking effective ways to delay or ameliorate MCI in order to prevent AD. Cognitive training is considered a promising way to delay the decline of cognitive function, and indeed, promising results have been observed for MCI (Jean et al., 2010; Hyer et al., 2016; Lin et al., 2017). Recent meta-analyses have identified its significant cognitive outcomes in multidomain and lifestyle approaches for individuals with MCI (Sherman et al., 2017). Also, cognitive training is a more effective and economical method compared to pharmacological approaches (Dresler et al., 2013).

Cognitive training is a set of training programs designed by researchers, including standardized training instructions and training tasks, and each task is targeted at improving one or several specific cognitive functions. Since the 1990s, researchers have attempted to improve the cognitive function of elder people by using cognitive training. The initial training mode is mainly based on the paper-pencil intelligence tests and the techniques of enhancing memory. For instance, researchers provide the participants with some scattered numbers and letters and ask them to connect the numbers and letters in alphabetical order by drawing lines (Jobe et al., 2001). With the popularity of electronic devices, standardized computer-based cognitive training programs have been gradually developed. Computer-based cognitive training has advantages of high efficiency and high sensitivity, which means its training materials can be coded in the computer and automatically presented to the participants, and the degree of training difficulty can be self-adaptive according to the performance of the participants (Dresler et al., 2013). Also, computer-based cognitive training can contribute to the moderation of cognitive disorders in dementia, particularly in the early stages of AD, as have been confirmed by several researches (Preece and Maloney-Krichmar, 2003; Savitch and Zaphiris, 2006).

Although the cognitive trainings are able to improve some specific cognitive functions, training every domain of cognition is difficult and time-consuming. Thereby, cognitive decline in the elderly is still widespread. In this case, the effect of training on non-trained cognitive functions (i.e., the transfer effect) should be discussed. The transfer effect refers to the ability that individuals can use the knowledge and skills learned in one scenario to achieve different goals in other scenarios. And it can be differentiated into near-transfer effects (post-training improvement in tasks similar to the training tasks) and far-transfer effects (post-training improvement in tasks that are different from the training tasks in nature or in appearance; Barnett and Ceci, 2002). Far-transfer effects occur when two different tasks share an underlying processing component and neuroanatomical areas or neural circuits (Jonides, 2004).

According to the transfer effects, researchers attempted to transfer the effects of cognitive training to other cognitive abilities to help MCI elder people adapt to their daily lives and solve corresponding problems. Researchers trained hearing processing speed of the MCI elderly and found that the participants not only showed faster processing speed but also had a higher accuracy rate in the auditory signal detection task. Besides, the participants also showed enhancement in memory (Anderson et al., 2013). More surprisingly, cognitive training also showed a transfer effect in physical activities (Verghese et al., 2010; Smith-Ray et al., 2014, 2015). For instance, after the Stroop training, MCI participants not only showed enhancement in working memory and executive functions but also had better performance in terms of physical balance (Li et al., 2011). However, there are also studies that did not find transfer effect after training cognitive functions (Dosher and Lu, 1998). And the sustainability of transfer effects after cognitive training is barely discussed.

There is no doubt that to explore the transfer effects in cognitive training is meaningful and imperative. On one hand, if the transfer effects are accessible and sustainable, the efficiency of cognitive training will be greatly improved. In other words, elderly people with MCI only need to receive a small amount of training to get multi-domain benefits. On the other hand, the transfer effects of cognitive training can overcome many training obstacles, such as disability and physical aging. That means researchers are able to help the bedridden elder people (e.g., people with stroke) improve their abilities which are hard to be trained (e.g., ability of daily living) by the transfer effects.

### Aim of Study

**Figure 1:**
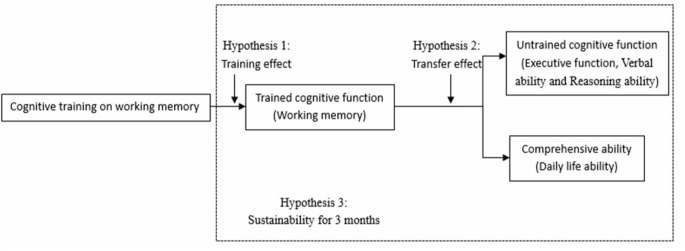
Hypothesis of cognitive training model.

The main objective of this study is to examine whether single-domain cognitive training (working memory training) has a transfer effect on un-trained cognitive functions, based on the effectiveness of cognitive training. The reason of choosing working memory is because it is a fundamental cognitive function that underpins other complex cognitive functions (Morrison and Chein, 2010). And the cognitive functions might be affected as follows (see Figure 1).

Hypothesis 1 (training effect): working memory would significantly improve after cognitive training.

Hypothesis 2 (transfer effect): the un-trained cognitive function (executive function, verbal ability and reasoning ability) and comprehensive ability (ability of daily living) would significantly improve after training.

Hypothesis 3 (sustainability): the training effect and the transfer effect can maintain for at least 3 months.

## Method

### Participants

The present study was approved by the Human Subjects Review Committee of Zhejiang University. Participants consisted of MCI elderly, over 60 years old, living in Nanxing Street, Shangcheng District, Hangzhou City, and participants were recruited *via* holding recruitment sessions at older adult communities.

The inclusion criteria were as follows:

1. Age ≥60 years old.
2. With no significant visual or auditory impairment.
3. The Montreal Cognitive Assessment (MoCA, <26 when education level >12 years or MoCA <25 when education level ≤12 years).
4. Meeting the MCI diagnostic criteria of the National Institute of Neurological Disorders and Stroke Alzheimer Disease and Related Disorders (NINCDS-ADRDA; McKhann et al., 1984).
5. Noticed and freely to give informed consent.

The exclusion criteria were as follows:

1. Meeting the dementia diagnostic criteria of the Diagnostic and Statistical Manual of Mental Disorders, fifth edition (DSM-V; American Psychiatric Association, 2013) and NINCDS-ADRDA.
2. Taking antipsychotics, or stopping for less than 3 months.
3. Participating in other cognitive training projects.
4. Refusing to participate in the study.

**Figure 2:**
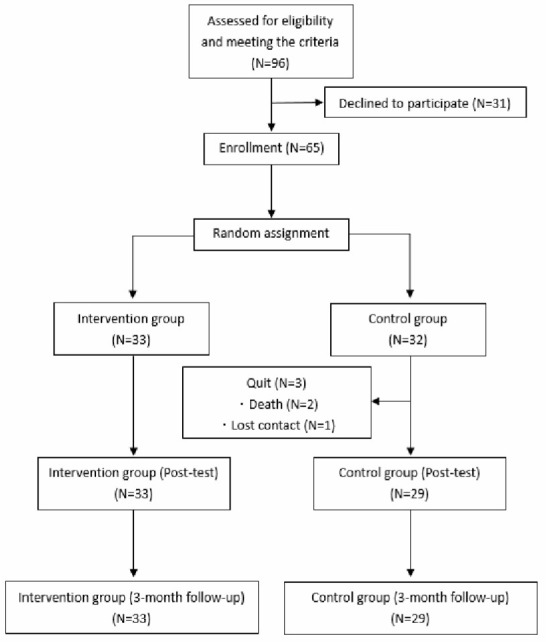
Research procedure.

After recruitment, there were 96 individuals who met the criteria while 31 participants were declined to participate because of lack of time for training or fulfilling 3-months follow-up. The enrolled participants (*N* = 65) were randomly assigned to a cognitive training group (*N* = 33) or a control group (*N* = 32). All participants had signed up the informed consent and committed not to attend any other cognitive training activity during the present study. If an individual was on medication (i.e., memantine or cholinesterase inhibitors), it was required to have no changes in dosage from the 3 months prior to recruitment to the end of the present study except in extreme circumstances. During the cognitive training, one participant fell off (lost contact), and two participants in the control group discontinued the study due to death (see in Figure 2).

According to the results of independent-sample *t*-test and *χ*^2^ test, there was no significant difference in the population and sociological variables between the two groups (see Table 1).

**Table 1:** Demographic information of cognitive training group and control group.

Variables	Cognitive training group (*n* = 33)		Control group (*n* = 29)		*t/χ*^2^
	*M/%*	*SD*	*M/%*	*SD*	*p*
Age	81.82	11.28	80.72	9.91	0.69
65–69	21.21%		20.69%		0.62
70–79	21.21%		20.69%		
80–89	30.30%		44.83%		
90–	27.27%		13.79%		
Gender (female)	87.88%		96.55%		0.22
Education (year)					
Illiteracy	42.42%		41.38%		0.79
1–6	18.18%		13.79%		
7–9	21.21%		24.14%		
10–	18.18%		20.69%		

### Procedures

The cognitive training group received a cognitive training of 8 weeks, 2 times/week for 40–60 min (Ross et al., 2018). The control group was treated with mental leisure activities (MLA) to simulate participants’ everyday mental activities and entertain them to prevent dropping out. Each time the cognitive training group received training, the researchers or community workers organized the control group to conduct activities (Lin et al., 2016). The activities mainly included watching TV dramas, watching news, blood pressure measurement, and chatting, etc. The present study collected cognitive evaluation data at three time-points, before cognitive training (T1), after cognitive training (T2), and 3-month follow-up (T3). The flowchart is presented in Figure 2.

### Cognitive Training

The present study used computer programs for cognitive training and mainly focused on the training of working memory, including “graphic/image/information delay matching,” “object tracking,” “memory/attention composite task.” All games were developed by Zhejiang University and Nanjing Zhihui Education Technology Company, Limited. The cognitive training contains four main tasks:

1. The graphic/image delay matching task: first, participants were asked to remember the picture(s) presented (one picture or more) on the screen (including simple shapes, landscape pictures). Then in the recognition phase, participants were asked to determine whether the new image has been studied before.
2. The information delay matching task: it required the participant to remember the photos of the characters presented on the screen, as well as the basic information related, and in the subsequent testing phase participants were asked to match the person’s photo and the corresponding individual information;
3. The object tracking task: it required participants to pay attention to the trajectory of one or more targets (the target can be a simple graphic or a physical picture) moving on the screen. After a period of time, the object disappears, and participants were asked to select the previous position of target object before it disappears;
4. Memory/Attention Composite task: it presented a scene containing one object 1 and multiple objects 2 and required participants to pay attention and click on the position of object 1 on the screen while remembering the number of objects 2. Both object 1 and objects 2 are simple graphic or physical objects. After clocking object 1, objects 2 immediately disappeared, then participants were required to report the number of objects 2.

### Measures

Basic demographic information was recorded, including gender (male/female), age and education level (1 = illiterate; 2 = primary school degree; 3 = junior high school degree; 4 = high school degree and above; Appendix 1).

Cognitive assessments focused on working memory, executive function, reasoning ability, verbal ability, ability of daily living. In the present study, three subtests of digit span, digital symbol conversion and similarity test in the Wechsler Adult Intelligence Scale-Fourth Edition of Chinese version (WAIS–IV; Wang et al., 2013) were used to evaluate working memory, executive function (conversion ability, untrained) and reasoning ability (untrained; Appendices 4–6). The Weng and Huang's (2014) study was used to evaluate the ability of daily living (Appendix 3). In addition, Montreal Cognitive Assessment (MoCA; Hongji et al., 2014; Razali et al., 2014; Gan et al., 2017) was used to evaluate comprehensive cognitive function, and its subtest (i.e., the verbal fluency test, VFT) was used to evaluate the verbal ability (Appendix 2).

In order to avoid the impact of operational familiarity caused by computer training program, all cognitive assessments were conducted in the form of paper and pencil.

### Analyses

The data for the present study were analyzed by IBM SPSS 21.0 and Mplus Version 8.2.

1. The independent-sample *t*-test and *χ*^2^ test were used to compare the baseline data of the cognitive training group with that of the control group.
2. The analysis of covariance (ANCOVA) was used to test the training effect. The data of cognitive assessments (T2) was the dependent variable, and the grouping condition was the independent variable while controlling for age, gender and baseline data (T0).
3. Mediating effect model (bootstrap analyses, bootstrap = 5,000) was used to test the transfer effect of cognitive training on un-trained cognitive functions and comprehensive ability, with working memory as the mediator.
4. Paired sample *t*-tests (T2-T3) were used to test the maintenance of training effect. And the ANCOVA was also used to test the long-term effect. The data of cognitive assessments (T3) was the dependent variable, and the grouping condition was the independent variable, while age, gender and baseline data (T1) were controlled for.
5. The value of *α* is 0.05, and the value of significance *p* is a two-sided probability.

## Results

### Training Effect

By independent-sample *t*-test and *χ*^2^ test, there were no significant differences in comprehensive cognitive function (MoCA), working memory (forward and backward digit span), executive function (digital symbol conversion), verbal ability (VFT), logic reasoning ability (the similarity reasoning test), ADL between the cognitive training group and the control group at baseline (see Table 2A).

**Table 2A:** Three time-points (baseline, post-training and 3 months after training) data of cognitive training group and control group.

Variables	Baseline					Post-training				3-months follow-up			
	Cognitive training group		Control group		*t*/χ^2^	Cognitive training group		Control group		Cognitive training group		Control group	
	*M/%*	*SD*	*M/%*	*SD*	*p*	*M/%*	*SD*	*M/%*	*SD*	*M/%*	*SD*	*M/%*	*SD*
MoCA	17.45	4.65	18.41	3.40	0.36	18.09	4.71	17.86	3.32	18.12	4.79	17.38	3.42
Digit span
Forward	4.42	1.25	4.03	1.12	0.20	4.55	1.23	3.76	1.12	4.33	1.24	3.79	1.15
Backward	2.45	0.94	2.69	0.97	0.34	2.73	0.91	2.52	0.91	2.67	0.82	2.41	1.02
Digital symbol conversion	19.03	8.12	21.45	7.78	0.24	21.48	6.70	20.10	8.03	20.48	6.28	20.17	8.12
VFT	8.94	3.03	9.59	2.71	0.38	9.06	2.68	9.66	2.76	9.15	2.46	9.52	2.59
Reasoning based on similarity	10.61	4.96	11.17	3.96	0.62	11.06	4.44	11.55	4.37	10.79	5.01	11.24	3.99
ADL	24.06	11.72	22.72	11.58	0.65	23.79	11.31	23.07	11.16	24.00	11.81	23.41	11.74

The ANCOVA was used to control the baseline, age, gender and education level of the participants. After the cognitive training (T2), forward digit span (*F*_(1,56)_ = 12.36, *p* < 0.01, *χ*^2^ = 0.18), backward digit span (*F*_(1,56)_ = 6.93, *p* < 0.05, *χ*^2^ = 0.11) and digital symbol conversion test (*F*_(1,56)_ = 17.38, *p* < 0.001, *χ*^2^ = 0.24) showed significant grouping differences. The difference in MoCA scores between the two groups was not significant after cognitive training (*F*_(1,56)_ = 3.79, *p* = 0.058, *χ*^2^ = 0.06). VFT, similarity reasoning test, and ADL scores were not significantly different (see Table 2B).

**Table 2B:** Analysis of covariance (ANCOVA) data between cognitive training group and controlled group in T2.

Variables	*F*	*p*	*η*^2^
MoCA	3.79	0.058	0.063
Digit span			
Forward	12.363	0.001	0.181
Backward	6.929	0.011	0.110
Digital symbol conversion	17.381	0.000	0.237
VFT	0.158	0.693	<0.01
Reasoning based on similarity	<0.01	0.998	<0.01
ADL	1.585	0.213	0.028

### Maintenance of Training Effect

**Figure 3:**
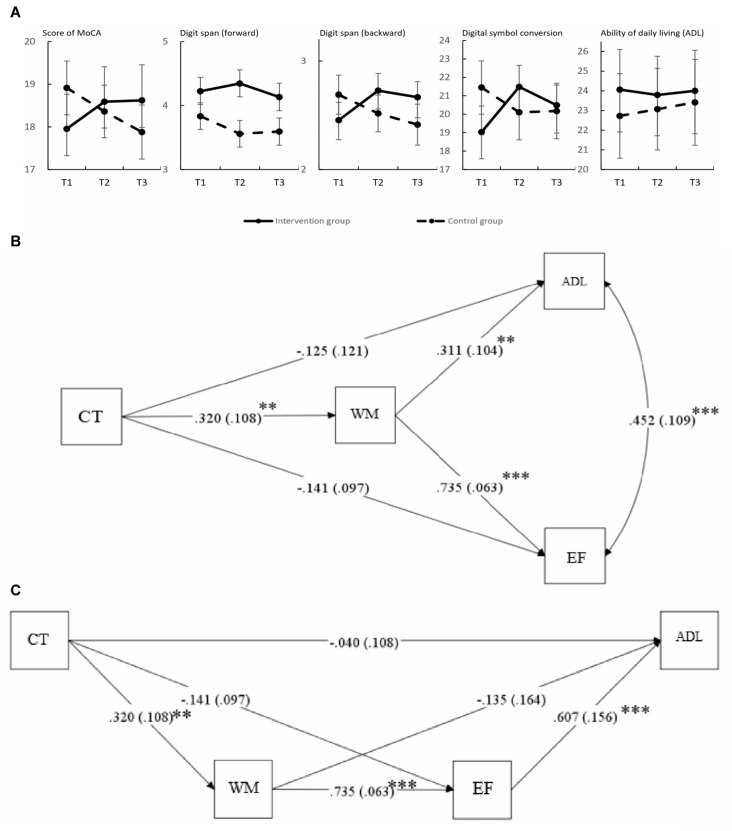
**(A)** Evaluation score in T1, T2 and T3. From left to right, the ordinates represent the score of Montreal Cognitive Assessment (MoCA), digit span (forward), digit span (backward), digital symbol conversion and ability of daily living (ADL). **(B)** Mediating effect model of cognitive training (CT represents cognitive training, WM represents working memory, EF represents excusive function and ADL represents ability of daily living; **p < 0.01, ***p < 0.001). **(C)** Mediating effect model of cognitive training (considering the relationship between executive function and ability of daily living; **p < 0.01, ***p < 0.001).

The scores of cognitive function and life ability at three times (T1, T2 and T3) can be seen in Figure 3A. Among them, cognitive ability, such as working memory and executive function, had a decline trend after the cognitive training. However, the results of the paired sample *t*-tests showed that, except for the executive function (conversion; *p* = 0.01), there was no significant change in other cognitive abilities and life abilities at 3-month follow-up (T3) compared to the end of cognitive training (T2; see Table 2C). It is worth mentioning that the MoCA and ADL scores of the cognitive training group remained at a specific level from T2 to T3, but the MoCA and ADL (reverse scoring) scores of the control group continued to decrease. These results indicate that the cognitive training mainly focused on working memory did not improve global cognitive function and ability of daily living, but there might be a long-term transfer effect.

**Table 2C:** Training effect in T2 and T3 (paired sample *t*-tests, cognitive training group).

Variables	Score in T2 (*M ± SD*)	Score in T3 *p*	(*M ± SD*)
MoCA	18.09 ± 4.71	18.12 ± 4.79	0.94
Digit span			
Forward	4.55 ± 1.23	4.33 ± 1.24	0.09
Backward	2.73 ± 0.91	2.67 ± 0.82	0.33
Digital symbol conversion	21.38 ± 7.70	20.48 ± 6.28	0.01
ADL	23.79 ± 11.31	24.00 ± 11.81	0.68

In order to explore the delay effect, the ANCOVA was used to control the baseline, age, gender and education level of the participants. Comparing the data at 3-months follow-up (T3) with baseline (T1), MoCA (*F*_(1,56)_ = 17.164, *p* < 0.001, *χ*^2^ = 0.24), backward digit span (*F*_(1,56)_ = 13.894, *p* < 0.001, *χ*^2^ = 0.199), digital sign conversion (*F*_(1,56)_ = 12.821, *p* < 0.01, *χ*^2^ = 0.186) and ADL (*F*_(1,56)_ = 9.30, *p* < 0.01, *χ*^2^ = 0.142) showed grouping differences. Compared with the post-cognitive training (T2) data, MoCA and ADL showed a grouping effect (see Table 2D).

**Table 2D:** ANCOVA data between cognitive training group and controlled group in T3.

Variables	*F*	*p*	*η*^2^
MoCA	17.164	0.000	0.235
Digit span		
Forward	3.382	0.071	0.057
Backward	13.894	0.000	0.199
Digital symbol conversion	12.821	0.001	0.186
VFT	0.491	0.486	<0.01
Reasoning based on similarity	0.034	0.854	<0.01
ADL	9.298	0.003	0.142

### Transfer Effect

It is difficult for cognitive training to train only one cognitive ability, for example, memory training must involve the use of attention resources, and computer operations also train exclusive function. In this case, the results of the general linear model cannot distinguish between the transfer effect and the training itself. Therefore, a mediating effect model (bootstrap analyses) was used to explore the mediating effect of working memory on un-trained cognitive functions (executive function) and comprehensive ability (ability of daily living) after cognitive training, in order to verify the transfer effect. The results showed that cognitive training had a significant training effect on working memory ability (*β* = 0.320, *p* < 0.01), and the effect of working memory ability on ability of daily living (*β* = 0.311, *p* < 0.01) and executive function (conversion; *β* = 0.735, *p* < 0.001) was significant. The effects of cognitive training on ability of daily living (*β* = −0.125, *p* = 0.245) and executive function (conversion; *β* = −0.141, *p* = 0.147) were not significant (see Figure 3B). The results indicated that cognitive training had no significant direct impact on ability of daily living and executive function.

In the above model, there was a significant correlation between DLA and EF, which suggested that WM may not directly affect DLA, with EF as the mediator in the process. Thus, another bootstrap analysis was used to explore the mediating effect of EF on the relation between WM and DLA (see in Figure 3C). The results showed that cognitive training had a significant effect on working memory ability (*β* = 0.320, *p* < 0.01), and the effect of WM on EF (*β* = 0.735, *p* < 0.001) and EF on DLA (*β* = 0.607, *p* < 0.001) was significant. The effects of cognitive training on DLA (*β* = −0.040, *p* = 0.713), cognitive training on EF (*β* = −0.141, *p* = 0.147) and WM on DLA (*β* = −0.135, *p* = 0.412) were not significant.

## Discussion

The present study used cognitive training on the community MCI elderly, mainly focusing on working memory. Then cognitive abilities and ability of daily living were evaluated to test the cognitive training effect, its maintenance and transfer effect of the cognition training. The results showed that cognition training had a high degree of acceptance in the in-home MCI elderly population in urban communities, and the compliance in the cognitive training process was satisfactory. And the results also indicated that cognition training not only enhanced the performance of MCI elderly people in working memory tests but also had a significant transfer effect in executive function and ability of daily living.

In addition, the MCI elderly people in communities showed varying degrees of decline in memory, attention, executive function, verbal ability, and abstract thinking ability, among which the decline of memory function was the most notable. For instance, in the delayed recall test, even after the relevant cue clues were given by the tester, all MCI participants could not fully recall the five words previously studied. Therefore, the researchers believe that the decline of memory function is a vital risk factor of the transformation from MCI into dementia (Gao et al., 2011).

The present study supports the theory of “cognitive plasticity” (Willis and Schaie, 2009), which embodies in the cognition improvement brought by the training. In the digit span task (forward and backward), the cognitive training group and the control group showed significant differences after the cognitive training. The above two tasks (forward and backward) mainly examined working memory ability of the participants, which was also the cognitive ability that the training mainly focused on. The results indicated that the training not only improved the performance of participants in the same type of test but also improved other cognitive function of the participants to complete relevant tasks. It also showed that, for the working memory capacity of MCI elderly people, cognitive training has played a role in delaying the decline, which was also proved by the previous studies (Dannhauser et al., 2014; Ross et al., 2018).

According to the results of the MoCA, MoCA is a very credible measurement of the cognitive functions for MCI elderly people, therefore it can effectively reflect the changes in the overall cognitive functions before and after the cognitive training (Nasreddine et al., 2005). The cognitive training used in the present study could effectively delay the decline of overall cognitive ability for MCI elderly people, which was supported by the result that the MoCA score tends to be stable. However, the improving effect on overall cognitive ability was not significant. The MoCA scores of the training group did not exceed the critical points of cognitive health standards in the present study.

The mediation model of the research results mostly excluded the possibility that training directly enhanced executive function, and supported the hypothesis that cognitive training has a transfer effect. Cognitive training has a significant transfer effect on executive function (conversion ability; Smith-Ray et al., 2015). The transfer effect of the participants on the digital symbol conversion task in the present study not only existed after cognitive training but also remained significant after 3-months follow-up. In addition to examining the executive functions (conversion), the digital symbol conversion task also examined the memory ability of the participants. For example, during the task, the subject needed to repeatedly compare the digital symbol conversion example, and then memorize the converted symbol and then wrote it on the answer sheet. And the memory ability was the training cognitive function of the present study, so it was more likely that the transfer effects showed up (Hausdorff et al., 2005). At the same time, some researchers believe that visual cognitive functions are mainly dominated by the frontal and parietal-related visual cortex, while tasks related to task execution and outcome feedback are mainly dominated by the prefrontal cortex (Thorpe et al., 1996). There is a large crossover between the two regions, so the use of visual cognitive training also has an impact on executive function. This sort of transfer effect may be called the “close-transfer effect” (Yogev et al., 2005).

The two tasks of verbal fluency and similarity reasoning examined the extraction of long-term memory and the ability to summarize abstract things. They belong to complex advanced cognitive functions and are more dependent on the crystal intelligence of the participants. In the present study, no relevant training transfer effect was found. Some researchers have found that through the inference game training, the summary ability of the participants was improved, so as the letter fluency (Ross et al., 2018). It has also been found that training for reasoning ability can improve the execution function in conversion (Schulz and Jobe, 2001). Combined with the results of this study, this effect may be one-way irreversible.

Apart from improvement in the untrained cognitive ability, the present study also found that cognitive training on working memory can also improve the ability of daily living of MCI elderly people. A significant difference in ADL scores was found at T3 (3-month follow-up). As shown in Figure 3A, the cognitive training group had a small downward trend after cognitive training, with the overall level remained stable. At three time points, scores of the control group showed an upward trend. The effect of cognitive training was not significant in the post-cognitive training test, which might be caused by insufficient training (Edwards et al., 2005; Seidler et al., 2010). Also, the present study found the cognitive training on WM improved ADL through the mediation of EF (see in Figure 3C). The ability to enhance physical activity through cognitive training (including *in situ* rotation, grip strength, etc.,) had a mediating effect on executive function, which was called “far-transfer effect” (Adams et al., 2005). However, the ADL assessed by this study is a complex comprehensive capability, so the existence of “far-transfer effect” in this process remains to be further verified.

Participants in the cognitive training group showed a maintenance effect on the MoCA and digit span (forward and backward) tasks, and there was no significant difference between post-cognitive training and 3-month follow-up (paired sample *t*-tests). In the data of 3-months follow-up (T3), the scores of the cognitive training group’s digital symbol conversion task decreased significantly, which reflected a decline in executive function. The effect of memory loss over time was significant in MCI (Nittrouer and Lowenstein, 2015). Thus, the reason which can interpret the results might be that executive function (conversion) became more pronounced as memory declines. Compared to the digit span (forward), the decline in the digit span (backward) score was slower. That might be due to the fact that digit span (backward) task not only examines the working memory capacity but also examines the spatial psychological rotation ability which requires visual space ability. And visual space ability was one of the main contents of visual cognitive training adopted in the present study. Some researchers have found that the training of spatial attention ability can improve the trainee’s working memory (digit span test score; Nasreddine et al., 2005). Therefore, the backward memory maintained better. Some researchers also believe that the abilities of forward and backward digit span are not developed simultaneously in childhood, because the backward task is relatively more complex. Thus, the decline of the forward memory ability is more significant in elderly people (Myerson et al., 2003).

There are still some limitations in the present study. First, due to research resource constraints, the total sample size of the study was small, and most of the participants were female (*N* female = 57). According to a 2017 review of cognitive trainings (Shah et al., 2017), only 11 of the 26 studies had a total sample size of less than 62 (the present study). Although the conclusions of the present study are basically consistent with previous research results, and the assumptions of the effectiveness and transfer effects of cognitive training on community MCI elderly are validated, future researches need to expand the sample size in order to eliminate the interference of irrelevant factors. Second, the present study only discussed conversion in executive function, however, the execution function is a complex cognitive function which also contains refresh and suppression. Future research can further explore the transfer effect on execution function more comprehensively. Third, the previous researches were based on theories of neural plasticity, but the results of the present study did not explain the impact of visual cognition training on the brain structure of MCI elderly. That is, in-depth study in neuroimaging is required to explore whether there are structural changes in synapses and neural networks, or changes in gray matter density and blood oxygen balance in related brain regions. Finally, the research used objective indicators such as standard cognitive psychology tests. A study in Hong Kong, China, pointed out that cognitive trainings can affect the subjective feelings of the elderly on their own health status (Kwok et al., 2013), and subjective well-being is an important evaluation index in the construction of home-based care environment. Therefore, it is necessary to add relevant subjective psychological rating scales or interviews in order to more comprehensively evaluate the effects of cognitive trainings.

## Conclusion

**Figure 4:**
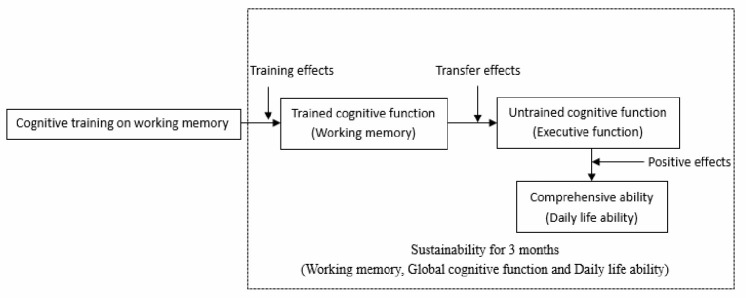
Possible mechanism of cognitive training.

The present study acquires a schematic diagram of the revised model of the mechanism of visual cognition training (see in Figure 4). The specific conclusions are as follows:

1. Cognitive training on working memory is able to delay the decline of MCI’s working memory, which effect will transfer to other untrained cognitive functions (execution functions);
2. The effect of cognitive training can not only transfer to other untrained cognitive functions (execution functions) but also affect the comprehensive ability (ability of daily living) positively;
3. The effect of cognitive training on comprehensive cognitive function, working memory and ability of daily living of MCI can be maintained for at least 3 months.

## Data Availability

All datasets generated for this study are included in the manuscript and/or the Supplementary Files.

## Ethics Statement

This study was carried out in accordance with the recommendations of the Human Subjects Review Committee of Zhejiang University with written informed consent from all subjects. All subjects gave written informed consent in accordance with the Declaration of Helsinki. The protocol was approved by the Human Subjects Review Committee of Zhejiang University.

## Author Contributions

WW and JL provided the idea of the article. WW was responsible for writing and editing this article. SC directed the entire article as the corresponding author. JX, TZ, YJ and JW helped with experimental training and data processing.

## Conflict of Interest Statement

The authors declare that the research was conducted in the absence of any commercial or financial relationships that could be construed as a potential conflict of interest.
