# Supplementary material for: The Transfer Effects of Cognitive Training on Working Memory Among Chinese Older Adults With Mild Cognitive Impairment: A Randomized Controlled Trial
Source: Front Aging Neurosci. 2019 Aug 14;11:212. doi: 10.3389/fnagi.2019.00212 (PMC6702334; doi:10.3389/fnagi.2019.00212)
Supplement: Supplementary file 1 [file Table_3.docx]

**Appendix**

**Appendix 1：Basic demographic information**

1.您的年龄是：__________岁？ （性别：1.男；2.女）

2.您的文化程度是：

（1）未受过正规教育；

（2）小学水平（1-6年）；

（3）初中或初中中专水平（7-9年）；

（4）高中或高中中专水平（10-12年）；

（5）大专及以上水平（12年以上）。

3.日常活动情况

从事家务等劳动的频率如何？

（1）几乎每天；

（2）不是每天，每周至少一次；

（3）不是每周，每月至少一次；

（4）不是每月，偶尔会做；

（5）几乎不做。

从事休闲社会活动的频率如何？

（1）几乎每天；

（2）不是每天，每周至少一次；

（3）不是每周，每月至少一次；

（4）不是每月，偶尔会做；

（5）几乎不做。

4.是否有慢性病？ （1.是；2.否）

**Appendix 2：Montreal Cognitive Assessment（MoCA）**

**当场计分**：受教育程度**>12**年者，分数**<26**，入组；受教育程度**<12**年者，分数**<25**，入组。


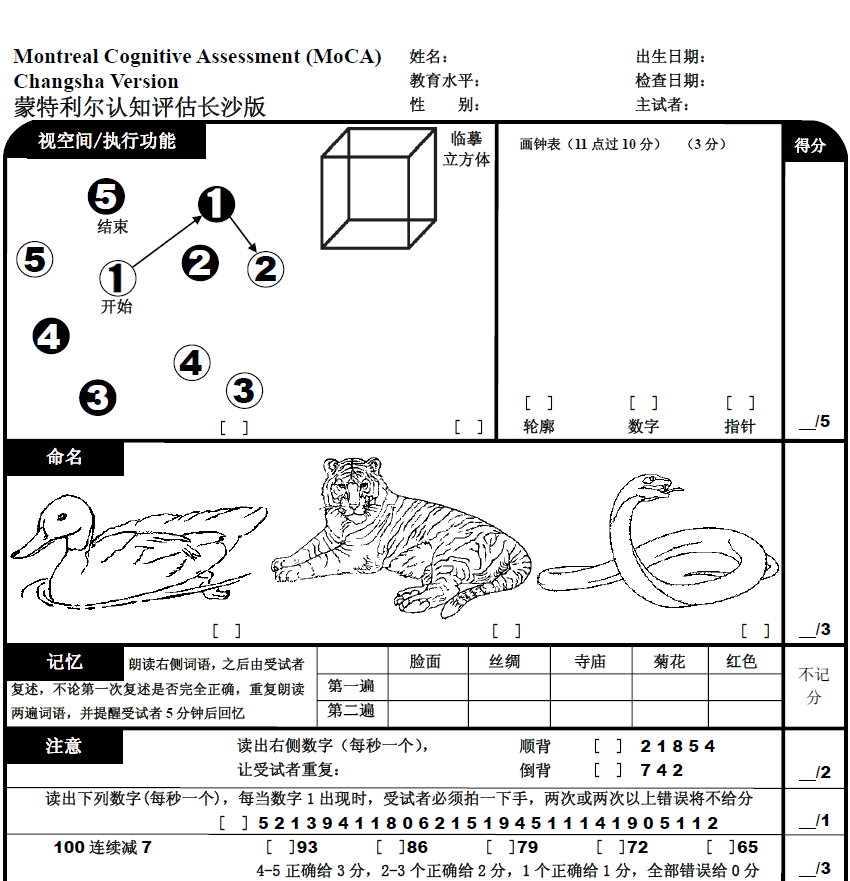

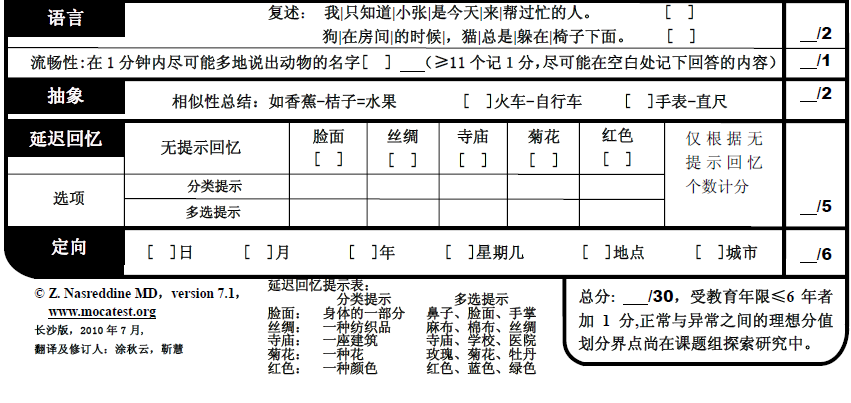


**Appendix 3：Ability of daily living scale（ADL）**

| 题目 | 评分 | | | |
| --- | --- | --- | --- | --- |
|  | 完全可以自理 | 有些困难 | 需要帮助 | 完全无法自理 |
| 1. 乘坐公交车 | 1 | 2 | 3 | 4 |
| 2. 行走 | 1 | 2 | 3 | 4 |
| 3. 做饭菜 | 1 | 2 | 3 | 4 |
| 4. 做家务 | 1 | 2 | 3 | 4 |
| 5. 吃药 | 1 | 2 | 3 | 4 |
| 6. 吃饭 | 1 | 2 | 3 | 4 |
| 7. 穿衣服 | 1 | 2 | 3 | 4 |
| 8. 梳头、刷牙等 | 1 | 2 | 3 | 4 |
| 9. 洗衣服 | 1 | 2 | 3 | 4 |
| 10. 洗澡 | 1 | 2 | 3 | 4 |
| 11. 购物 | 1 | 2 | 3 | 4 |
| 12. 上厕所（大小便） | 1 | 2 | 3 | 4 |
| 13. 打电话（拨号） | 1 | 2 | 3 | 4 |
| 14. 个人理财 | 1 | 2 | 3 | 4 |

**Appendix 4：digit span（forward and backward，** **WAIS-IV）**

| 正背 | | | 倒背 | | |
| --- | --- | --- | --- | --- | --- |
| 3 | 5-8-2  6-9-4 |  | 2 | 2-4  5-8 |  |
| 4 | 6-4-3-9  7-2-8-6 |  | 3 | 6-2-9  4-1-5 |  |
| 5 | 4-2-7-3-1  7-5-8-3-6 |  | 4 | 3-2-7-9  4-9-6-8 |  |
| 6 | 6-1-9-4-7-3  3-9-2-4-8-7 |  | 5 | 1-5-2-8-6  6-1-8-4-3 |  |
| 7 | 5-9-1-7-4-2-8  4-1-7-9-3-8-6 |  | 6 | 5-3-9-4-1-8  7-2-4-8-5-6 |  |
| 8 | 5-8-1-9-2-6-4-7  3-8-2-9-5-1-7-4 |  | 7 | 8-1-2-9-3-6-5  4-7-3-9-1-2-8 |  |
| 9 | 2-7-5-8-6-2-5-8-4  7-1-3-9-4-2-5-8-6 |  | 8 | 9-4-3-7-6-2-5-8  7-2-8-1-9-6-5-3 |  |
| 10 | 5-2-7-4-9-1-3-7-4-6  4-7-2-5-9-1-6-2-5-3 |  | 9 | 6-3-1-9-4-3-6-5-8  9-4-1-5-3-8-5-7-2 |  |
| 11 | 4-1-6-3-8-2-4-6-3-5-9  3-6-1-4-9-7-5-1-4-2-7 |  | 10 | 6-4-5-2-6-7-9-3-8-6  5-1-6-2-7-4-3-8-5-9 |  |
| 12 | 7-4-9-6-1-3-5-9-6-8-2-5  6-9-4-7-1-9-7-4-2-5-9-2 |  |  |  |  |

**Appendix 5：****Digital symbol conversion (WAIS-IV)**

| 1 |  | 2 |  | 3 |  | 4 |  | 5 |  | 6 |  | 7 |  | 8 |  | 9 |
| --- | --- | --- | --- | --- | --- | --- | --- | --- | --- | --- | --- | --- | --- | --- | --- | --- |
| ⎯ |  | ⊥ |  | コ |  | 𠃊 |  | 凵 |  | 〇 |  | ∧ |  | × |  | ＝ |

| **样本（练习）** | | | | | | | | | |  |  |  |  |  |  |  |  |  |  |  |  |  |  |  |
| --- | --- | --- | --- | --- | --- | --- | --- | --- | --- | --- | --- | --- | --- | --- | --- | --- | --- | --- | --- | --- | --- | --- | --- | --- |
| 2 | 1 | 3 | 7 | 2 | 4 | 8 | 1 | 5 | 4 | 2 | 1 | 3 | 2 | 1 | 4 | 2 | 3 | 5 | 2 | 3 | 1 | 4 | 6 | 3 |
|  |  |  |  |  |  |  |  |  |  |  |  |  |  |  |  |  |  |  |  |  |  |  |  |  |
|  |  |  |  |  |  |  |  |  |  |  |  |  |  |  |  |  |  |  |  |  |  |  |  |  |
| 1 | 5 | 4 | 2 | 7 | 6 | 3 | 5 | 4 | 2 | 8 | 5 | 4 | 6 | 3 | 7 | 2 | 8 | 1 | 9 | 5 | 8 | 4 | 7 | 3 |
|  |  |  |  |  |  |  |  |  |  |  |  |  |  |  |  |  |  |  |  |  |  |  |  |  |
|  |  |  |  |  |  |  |  |  |  |  |  |  |  |  |  |  |  |  |  |  |  |  |  |  |
| 6 | 2 | 5 | 1 | 9 | 2 | 8 | 3 | 7 | 4 | 6 | 5 | 9 | 4 | 8 | 3 | 7 | 2 | 6 | 1 | 5 | 4 | 6 | 3 | 7 |
|  |  |  |  |  |  |  |  |  |  |  |  |  |  |  |  |  |  |  |  |  |  |  |  |  |
|  |  |  |  |  |  |  |  |  |  |  |  |  |  |  |  |  |  |  |  |  |  |  |  |  |
| 9 | 2 | 8 | 1 | 7 | 9 | 4 | 6 | 8 | 5 | 9 | 7 | 1 | 8 | 5 | 2 | 9 | 4 | 8 | 6 | 3 | 7 | 9 | 8 | 6 |
|  |  |  |  |  |  |  |  |  |  |  |  |  |  |  |  |  |  |  |  |  |  |  |  |  |

- 测试时长90秒；
- 正确记1分，倒转符号记0.5分，10个以内记0分，10个以上按正确数计分。

**Appendix 6：Similarity test (WAIS-IV)**

指导语：

这里有若干对词语，请您看看它们之间有什么相类似的地方，也就是说，请把它们的共同点进行概括。

| 序号 | 词对 | 共同点 | 是否正确 | |
| --- | --- | --- | --- | --- |
| 1 | 斧头——锯子 | 木工工具（2分）；铁质（1分） | 是 | 否 |
| 2 | 狗——狮子 | 动物等（2分）；四脚等次要特征（1分） | 是 | 否 |
| 3 | 桔子——桃子 | 水果等（2分）；可食用等次要特征（1分） | 是 | 否 |
| 4 | 桌子——椅子 | 家具等（2分）；吃饭等特定功能（1分） | 是 | 否 |
| 5 | 帽子——袜子 | 衣物等（2分）；日用品或防寒等（1分） | 是 | 否 |
| 6 | 蛋——种子 | 孕育生命等（2分）；生物等宽泛概括（1分） | 是 | 否 |
| 7 | 北方——西方 | 方位等（2分）；气候温度等次要属性（1分） | 是 | 否 |
| 8 | 眼睛——耳朵 | 感官等（2分）；长在脸上等（1分） | 是 | 否 |
| 9 | 空气——水 | 生命必须（2分）；无色无味等次要特征（1分） | 是 | 否 |
| 10 | 诗——雕像 | 艺术品（2分）；纪念意义等（1分） | 是 | 否 |
| 11 | 表扬——处罚 | 使人进步等目的（2分）；一种评价（1分） | 是 | 否 |
| 12 | 木头——酒精 | 有机物等（2分）；可燃等次要属性（1分） | 是 | 否 |
| 13 | 苍蝇——树 | 生命（2分）；需要水等（1分） | 是 | 否 |
